# CO Methanation over NiO-CeO_2_ Mixed-Oxide Catalysts Prepared by a Modified Co-Precipitation Method: Effect of the Preparation pH on the Catalytic Performance

**DOI:** 10.3390/nano12152627

**Published:** 2022-07-30

**Authors:** Amar Bendieb Aberkane, María Pilar Yeste, Fayçal Djazi, Miguel Ángel Cauqui

**Affiliations:** 1 Laboratoire de Recherche sur la Physico-Chimie des Surfaces et Interfaces (LRPCSI), Faculté de Technologie, Département de Génie des Procédés, Université 20 Août 1955-Skikda, BP 26, Route d’El Hadaiek, Skikda 21000, Algeria; f.djazi@univ-skikda.dz; 2 Departamento de Ciencia de los Materiales e Ingeniería Metalúrgica y Química Inorgánica, Universidad de Cádiz, Campus Río San Pedro s/n, 11510 Cádiz, Spain; pili.yeste@uca.es

**Keywords:** nickel, ceria, CO methanation

## Abstract

In this study, a series of NiO-CeO_2_ mixed-oxide catalysts have been prepared by a modified co-precipitation method similar to the one used for the synthesis of hydrotalcites. The syntheses were carried out at different pH values (8, 9 and 10), in order to determine the influence of this synthetic variable on the properties of the obtained materials. These materials were characterized by using different techniques, such as TGA, XRD, ICP, N_2_ adsorption-desorption isotherms, H_2_ temperature-programmed reduction (H_2_-TPR), and electron microscopy, including high-angle annular dark-field transmission electron microscopy (HAADF-TEM) and EDS. The characterization results revealed the influence of the preparation method, in general, and of the pH value, in particular, on the textural properties of the oxides, as well as on the dispersion of the Ni species. The catalyst prepared at a higher pH value (pH = 10) was the one that exhibited better behavior in the CO methanation reaction (almost 100% CO conversion at 235 °C), which is attributed to the achievement, under these synthetic conditions, of a combination of properties (metal dispersion, specific surface area, porosity) more suitable for the reaction.

## 1. Introduction

CO methanation is a chemical process in which methane (CH_4_) is obtained from syngas (Equation (1)). It has attracted much attention in recent years, in relation to: (i) the removal of residual CO fractions in large ammonia production plants, or in hydrogen-rich gases feeding proton exchange membrane fuel cells (PEMFCs) [1,2], and (ii) the production of synthetic natural gas (SNG), in a process in which syngas is previously obtained by gasification of coal or biomass [3]. With the development of so-called ‘green’ hydrogen [4], CO methanation has also found a potential application in energy storage.

(1) $$\mathrm{CO}\left(g\right)+3H_{2}\left(g\right)\to {\mathrm{CH}}_{4}\left(g\right)+H_{2}O\left(g\right)\;\;{\Delta H}_{298}^{0}=-206\;\mathrm{kJ}/\mathrm{mol}$$

Considering both thermodynamic and kinetic factors, it is favoured under low temperature and high pressure, or high temperature experimental conditions. In recent decades, much research has focused on the development of stable, highly active and selective CO methanation catalysts. Due to their high activity and low price, Ni-based catalysts have often been proposed as the best option for activating the reaction. The effect of the support has also proved to be particularly important [5,6,7,8]. Thus, for example, Le et al. investigated Ni catalysts supported on different supports such as Al_2_O_3_, SiO_2_, TiO_2_, ZrO_2_ and CeO_2_ and determined that, of these, CeO_2_ was the most active for CO methanation [5]. As is well established, the catalytic properties of CeO_2_ are due to its unique oxygen storage capacity, its redox properties, and the possibility of exhibiting oxygen vacancies under a wide range of conditions.

CO methanation is a structure-sensitive reaction and, therefore, parameters such as the size of the metal particles significantly affect the activity and selectivity of the catalysts [9,10,11]. In reference to this, different synthetic approaches have been reported that allow influence over these parameters. For example, high surface area and active Ni/CeO_2_ catalysts were prepared, by using a surfactant-assisted co-precipitation method [12]. Hard-templated mesoporous NiO-CeO_2_ mixed oxides, with different Ni/Ce molar ratios, were also found very active and highly selective to CH_4_ [13]. Yan et al. [14] reported that the control of nucleation and crystal growth, induced by plasma decomposition of nickel precursor in Ni/SiO_2_, resulted in improved activity and enhanced coke resistance, compared with analogous systems prepared by thermal decomposition. Jiang et al. reported the preparation of a Ni/Al_2_O_3_ catalyst with an NiO loading of 40 wt% through a novel cation-anion double-hydrolysis strategy. The resulting Ni/Al_2_O_3_ catalyst showed high Ni dispersion with small particle size (<5 nm), despite the high metal loading. Upon promotion with Zr, the catalyst exhibited improved catalytic performance, mainly attributable to the high degree of Ni dispersion and the abundance of oxygen vacancies, which enhanced the adsorption and dissociation of CO [15]. Traditional methods, including impregnation, sol-gel, deposition-precipitation or hydrothermal synthesis, have also been applied [16,17,18,19]. Although all these synthetic procedures were aimed at improving Ni dispersion, some other parameters, such as support texture and metal-support interaction, have also been highlighted in these investigations as playing a key role in the catalytic performance of Ni for CO methanation; however, the relative influence of these parameters on the final behaviour of the material is often unclear.

In this paper, we report on the synthesis of a series of NiO-CeO_2_ mixed oxides prepared by using a modified co-precipitation method similar to the one used for the synthesis of hydrotalcites. Hydrotalcite-like materials are an important class of inorganic solids, having a structural similarity with the hydrotalcite mineral Mg_6_Al_2_(OH)_16_CO_3_·4H_2_O. They exhibit the general formula [M^2+^_1−*x*_M^3+^*_x_*(OH)_2_][A*^n^*^−^]*_x_*_/*n*_·*y*H_2_O, where M^2+^ and M^3+^ refer to divalent and trivalent metal cations, respectively (Ni^2+^ and Ce^3+^ in our case), and A*^n^*^−^ is a charge-compensating anion sandwiched between the cationic layers (CO_3_^2−^, OH^−^, etc.). The molar ratio [M^3+^/(M^2+^ + M^3+^)] normally varies between 0.2 and 0.4. As reported in a recent review by Kumari et al. [20], hydrotalcite-like materials (and mixed metal oxides derived from their calcination) exhibit unique characteristics for heterogeneous catalytic applications, such as thermal stability and the possibility of tailoring properties, such as basicity/acidity, specific surface area, or porosity. It is also likely that the interaction between M^2+^(Ni) and M^3+^(Ce) species differs from that obtained by conventional co-precipitation methods. The pH value has been used as a synthesis variable in order to determine its influence on the properties of the final product. Through the application of different characterisation techniques, results have been obtained that provide insight into the influence of parameters—such as the available Ni surface area, the Ni–CeO_2_ interaction, and the textural properties of these materials—on their performance in the CO methanation reaction.

## 2. Materials and Methods

### 2.1. Preparation of Catalysts

Ni–Ce mixed oxides with a calculated molar ratio Ni/Ce = 3 were obtained by co-precipitation following the hydrotalcite route at pH values of 8, 9, and 10 (±0.22).

Ni(NO_3_)_2_·6H_2_O [Sigma Aldrich, Schnelldorf, Germany, 98%] and Ce(NO_3_)_3_·6H_2_O, [Sigma Aldrich, Schnelldorf, Germany, 99%] were used as precursors. An aqueous solution of these precursors, with the appropriate Ni/Ce molar ratio (Ni(NO_3_)_2_·6H_2_O (0.6 M), Ce(NO_3_)_3_·6H_2_O (0.2 M), formed solution A. Na_2_CO_3_ (Cheminova International S.A, Madrid, Spain, 98%) was dissolved in water, to obtain the alkaline solution, B (0.16 M). Solution A was added dropwise to solution B under vigorous stirring, using a NaOH solution (2M) for pH control. After 24 h at room temperature, the precipitates were transferred to the reflux system and stirred at 85 °C for 24 h. The resulting gels were washed with pure water and ethanol several times to remove any excess salts. After filtration and washing/centrifugation, the solids were dried overnight in an oven at 100 °C. Finally, the samples were calcined in a muffle furnace at 600 °C for 1 h, with a heating ramp of 10 °C/min. The resulting materials were denoted as NiOCeO_2_-8, NiOCeO_2_-9, and NiOCeO_2_-10, where the numbers indicate the pH used in the synthesis.

### 2.2. Catalysts Characterization

Thermogravimetric analysis (TGA) was used to identify the optimum calcination temperature. The analyses were carried out using a thermogravimetric analyser (TA instruments Q50). The samples were heated in air to 900 °C at a rate of 10 °C/min.

Powder X-ray experiments were carried out on a Bruker D8 Advance A-25 diffractometer working in Bragg-Brentano geometry, using Cu Kα radiation (λ = 1.54 Å) as the radiation source. X-ray diffractograms were collected at room temperature over the 2 Theta range from 10 to 80°, with a stepwise increment of 0.02° and an acquisition time of 3 s at every angle. After the data collection, the XRD pattern analyses of all samples were identified in accordance with the reference patterns recorded in the JCPDS database. Scherrer and Bragg equations were used to estimate the crystallite size and lattice parameters.

The Ni content in the prepared catalysts was determined using inductively coupled plasma atomic emission spectroscopy (ICP-AES) (thermo elemental IRIS Intrepid model, Thermo Fisher Scientific, Waltham, MA, USA).

The S_BET_ (specific surface area) of the catalysts were measured by N_2_ adsorption-desorption isotherms at −196 °C, using an automatic volumetric system (Autosorb iQ3, Quantachrome Instruments, Boynton Beach, FL, USA). Prior to the measurement, the samples were degassed under vacuum for 2 h at 200 °C to remove physically adsorbed components and other adsorbed gases from the catalyst surface. The pore volume of the catalysts was obtained using the BJH method (Barret-Joyner-Halenda).

TPR studies were carried out on a Micromeritics AutoChem II 2920 instrument (Norcross, GA, USA), equipped with a thermal conductivity detector (TCD). For analysis, typically 50 mg of the sample was placed between two plugs of quartz wool in a tubular quartz reactor, then treated under a mixture of 5% H_2_/Ar at a flow rate of 50 mL/min in the temperature range 30–900 °C at a heating rate of 10 °C/min. The hydrogen consumption was quantitatively determined after calibration of the TCD response, using CuO as a standard.

The Ni dispersion, Ni metallic surface area and metallic Ni particle size of the prepared catalysts were estimated by hydrogen chemisorption at 35 °C (using a Micromeritics ASAP 2020C Instrument, Norcross, GA, USA). In a typical run, 150 mg of the sample were reduced in situ under H_2_ (5%)/Ar (60 mL/min) flow at 650 °C for 1 h. After reduction, the sample was kept in He flow at 650 °C for 1 h and then cooled in vacuum at 35 °C under He flow. The values for Ni dispersion (D_Ni_), metal surface area (S_Ni_) and Ni particle size (d_Ni_) were provided directly by the instrument software and estimated using Equations (2)–(4).

(2) $$D_{\mathrm{Ni}}\left(\%\right)=\frac{V_{m}{*W}_{\mathrm{Ni}}}{V_{\mathrm{molar}}{*M}_{c}}{*F}_{s}*100\times 100$$

(3) $$S_{\mathrm{Ni}}\left(m^{2}/g\right)=\frac{V_{m}{*N}_{A}{*F}_{s}{*A}_{\mathrm{Ni}}}{V_{\mathrm{molar}}}$$

(4) $$d_{\mathrm{Ni}}\left(\mathrm{nm}\right)=\frac{60{*M}_{c}}{\rho_{\mathrm{Ni}}{*S}_{\mathrm{Ni}}}$$

where V_m_ is the volume of hydrogen chemisorbed (cm^3^ g^−1^), V_molar_ is the molar volume of hydrogen (cm^3^ mol^−1^), M_c_ is the Ni percentage by weight, W_Ni_ is the atomic weight of Ni (g mol^−1^), A_Ni_ is the cross-sectional area of Ni atom (m^2^ atom^−1^), ρ_Ni_ is the Ni density (g cm^−3^) and Fs is the stoichiometry factor, which expresses the ratio between the number of active metal atoms and the number of adsorbate molecules (Ni_s_/H_2_). In this case, an Fs of 2 was assumed.

Scanning transmission electron microscopy (STEM) studies, both in high-angle annular dark-field (HAADF) and analytical X-ray energy dispersive spectroscopy (XEDS) modes, were performed in a JEOL 2010 F microscope (JEOL, Peabody, MA, USA), operating at 200 kV, with a structural resolution of 0.19 nm.

The NiOCeO_2_ catalysts were tested in CO methanation reaction under atmospheric pressure using a fixed bed reactor system. Typically, 50 mg of catalyst was diluted with 100 mg of SiC to avoid the generation of hotspots in the catalyst bed during the reaction. The catalyst/SiC mixture was loaded into the quartz reactor (internal diameter = 6 mm and length = 235 mm) and brought into contact with a feed composed of 1 mol% CO, 50 mol% H_2_, and 49 mol%He at a flow rate of 100 mL/min in the reaction temperature range 150–400 °C and gas hourly space velocity (GHSV) of 120,000 mL/g·h. The flow rate of inlet and outlet gases were controlled with a mass flow controller (MFC). To measure the reaction temperature, a thermocouple was placed in the catalyst bed. Prior to the catalytic activity tests, the samples were subjected to an activation treatment. This treatment consisted of a reduction in the catalyst, under 5% H_2_/Ar (100 mL/min) for 1 h at 650 °C and atmospheric pressure. A PFEIFFER quadrupole mass spectrometer, model Thermostar QME-200-D35614 (Pfeiffer Vacuum GmbH, Aßlar, Germany) was also used, to quantify the gases at the inlet and the outlet. The activity results are presented as a total percentage of the conversion of CO (and CH_4_ yield) as a function of the reaction temperature.

(5) $$\mathrm{CO}\;\mathrm{Conversion}\;(\%)=\frac{C_{\mathrm{COin}}-C_{\mathrm{COout}}}{C_{\mathrm{COin}}}*100$$

(6) $${\mathrm{CH}}_{4}\;\mathrm{Yield}\;(\%)=\frac{C_{\mathrm{CH}4\mathrm{out}}}{C_{\mathrm{COin}}}*100$$

here, C_COin_ represents the concentration of CO in the feed stream and C_COout_ (C_CH4out_), denote the concentration of CO and CH_4_ in the output stream.

## 3. Results

### 3.1. Catalysts Characterization

The TGA results (not shown) for our precursors indicated that above 600 °C no weight loss (corresponding to residual carbonates or nitrates species) was observed. This temperature was selected for calcination of the as-synthesised samples. Once calcined, the samples were characterized, to determine their chemical composition, structure and textural properties. The results of this characterization were discussed in detail elsewhere [21] and are gathered in Table 1. A CeO_2_ sample, prepared using the same synthetic method described for the mixed oxides (at pH = 8), was used as a reference in XRD experiments.

The fluorite-like structure of CeO_2_ and the cubic form of NiO were the only crystalline phases observed in the XRD patterns. The calculated lattice parameters are close to those reported for standard cubic NiO (4.177 Å) [22] and CeO_2_ (5.410 Å) [23]. However, in the case of CeO_2_ crystallites, a slight decrease of this parameter was observed as the pH of the preparation decreased, which can be interpreted, according to the literature, by the incorporation of Ni^2+^ species into the CeO_2_ lattice [22,24]. This incorporation will occur more noticeably in NiOCeO_2_-8 and NiOCeO_2_-9 samples.

As can be seen from results in Table 1, NiO crystallites are larger than CeO_2_ crystallites, and they become gradually smaller when the pH of the synthesis increases from 8 to 10. It is also important to note that the size of CeO_2_ crystallites is significantly smaller for NiO-CeO_2_ mixed oxides when compared to pure CeO_2_, suggesting that ceria crystallization is influenced by the presence of NiO.

**Figure 1:**
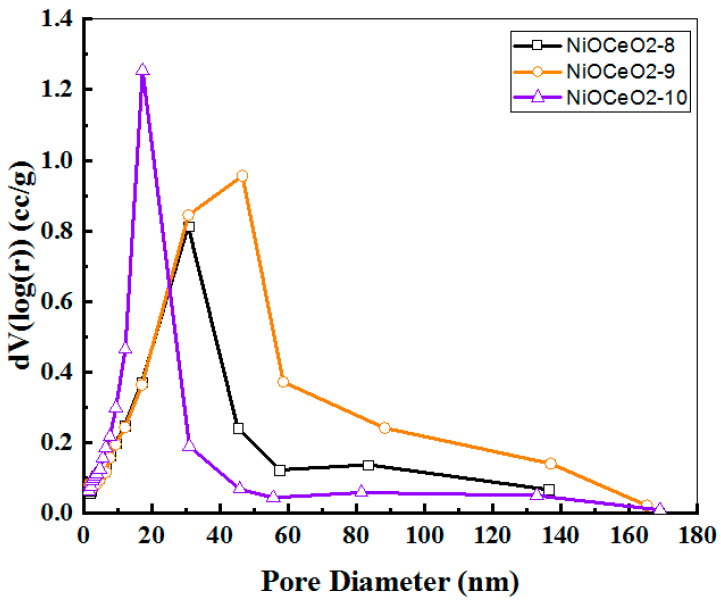
Pore size distribution obtained for NiOCeO_2_-8, NiOCeO_2_-9 and NiOCeO_2_-10 catalysts after calcination at 600 °C.

With regard to the results obtained from the N_2_ adsorption-desorption isotherms, these NiO-CeO_2_ catalysts are mesoporous and have a medium specific surface area, which increases as the pH of the preparation increases, in close agreement with the results for the crystallite sizes, as discussed above. Thus, the highest surface area was obtained for the NiOCeO_2_-10 sample. It is also noteworthy that the pore size distribution obtained for this oxide is much narrower (homogeneous) and shifted towards smaller pores, even though it has a much higher total pore volume than the samples obtained at lower pH (Figure 1). These results point to differentiated textural properties in the case of sample NiOCeO_2_-10, in relation to samples NiOCeO_2_-9 and NiOCeO_2_-8.

**Figure 2:**
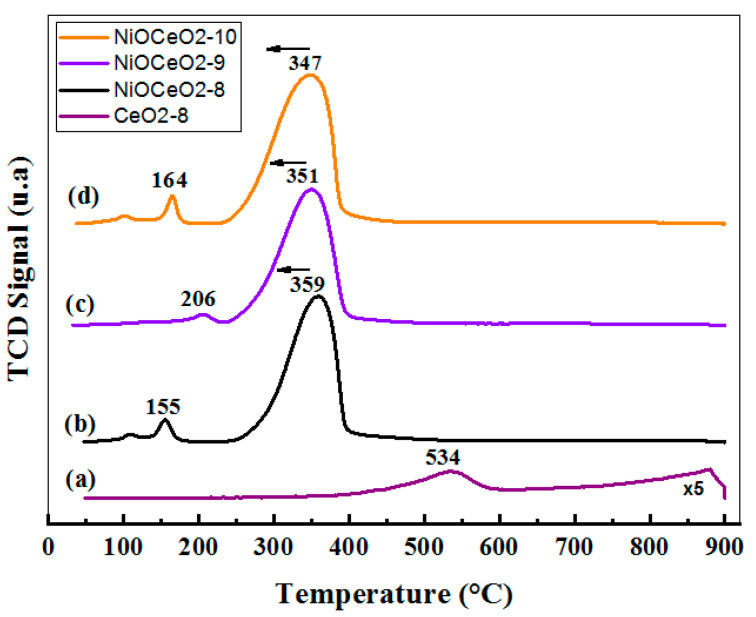
TPR profiles (H_2_ consumption) for samples CeO_2_-8 (**a**), NiOCeO_2_-8, (**b**) NiOCeO_2_-9 (**c**) and NiOCeO_2_-10 (**d**).

The ability of a material to activate the CO methanation reaction is often associated with its greater, or lesser, facility to form oxygen vacancies [15,25]. The temperature-programmed reduction (TPR) technique can provide relevant information in this respect. Figure 2 shows the traces monitored on the TDC detector corresponding to H_2_ consumption during TPR experiments. As can be seen in this figure, very similar reduction profiles were obtained for the three NiO-CeO_2_ mixed oxides. We observed an intense and asymmetric peak above 350–360 °C and also some small contributions at lower temperatures. Therefore, all reduction processes (including Ni^2+^→Ni and Ce^4+^→Ce^3+^) took place at temperatures below 400 °C, in contrast to the CeO_2_-8 sample, which was reduced at higher temperatures.

The position of the main peak is commonly correlated in the literature with the size of the NiO particles (larger particles are reduced at higher temperatures) and/or with the strength of the NiO-support interaction (stronger interactions result in higher reduction temperatures) [26,27]. In this case, as the pH value of the preparation increased, the peak shifted slightly, towards lower temperatures. This effect could be explained in terms of the decrease in crystallite sizes with pH, observed by XRD. However, the existence of a difference in the degree of interaction between NiO and CeO_2_, depending on the synthetic conditions, cannot be discarded. The peaks observed at low temperatures can be attributed to the reduction of oxygen adsorbed on the vacancies caused by the incorporation of Ni^2+^ within the CeO_2_ network, or to the reduction of surface adsorbed oxygen species easily accessible to H_2_ at low temperatures [19,26,28].

The Ni dispersion and some other related parameters (Ni particle size and Ni surface area) were estimated by H_2_ chemisorption. As can be seen in Table 2, the Ni dispersion values are very similar for the NiOCeO_2_-8 and NiOCeO_2_-9 catalysts, with only a very slight decrease observed for the latter. In contrast, raising the pH of the synthesis up to 10 resulted in a significant decrease of the dispersion by a factor of more than 3. Equivalent trends were obtained for Ni surface area, and particle size. These results are particularly interesting if we consider the N NiO-crystallite size values obtained via XRD (very similar for the three samples) and seem to indicate that the reduction treatment results in a much lower metallic surface area in the case of the NiOCeO_2_-10 sample.

According to these results, it would be reasonable to expect to see a significant influence exerted by the preparation pH on the catalytic behaviour of these oxides, and, in particular, it would be sample NiOCeO_2_-10 that, in principle, should offer the worst performance, because it is the one with the lowest metal surface available for the reaction.

**Figure 3:**
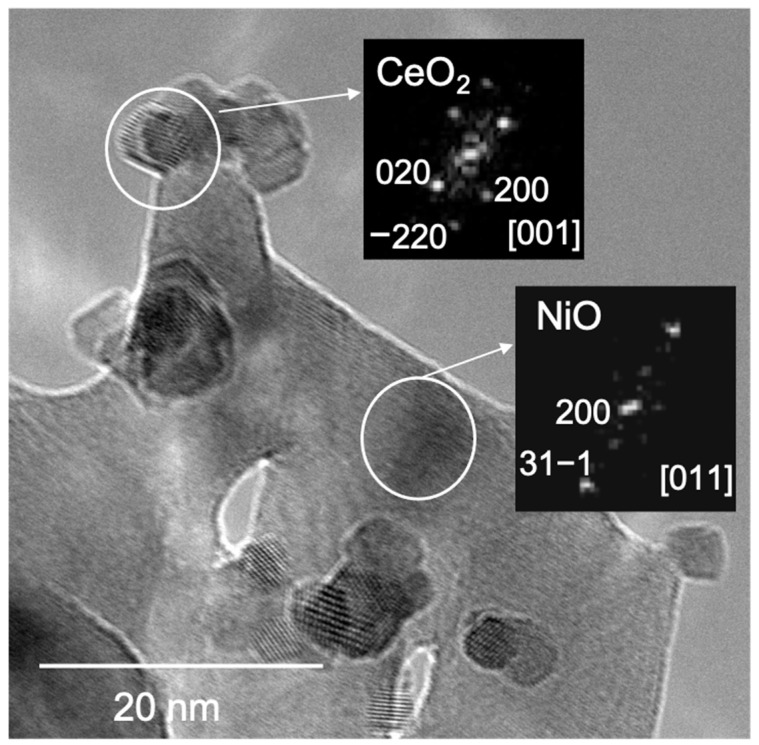
TEM image corresponding to the as-prepared NiOCeO_2_-8 catalyst.

**Figure 4:**
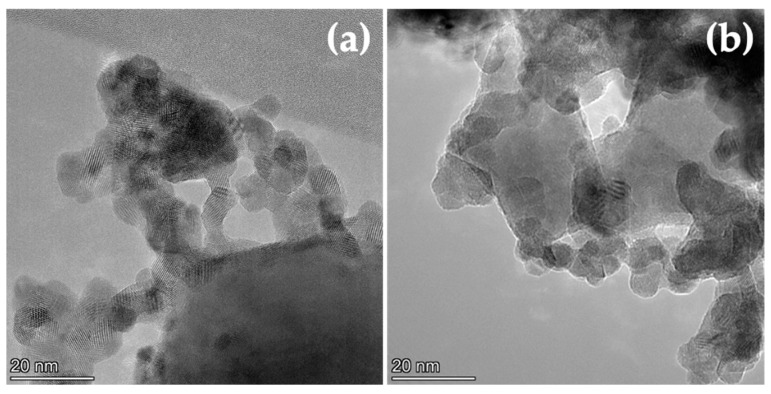
TEM images corresponding to the reduced NiOCeO_2_-8 (**a**) and NiOCeO_2_-10 (**b**) catalysts.

**Figure 5:**
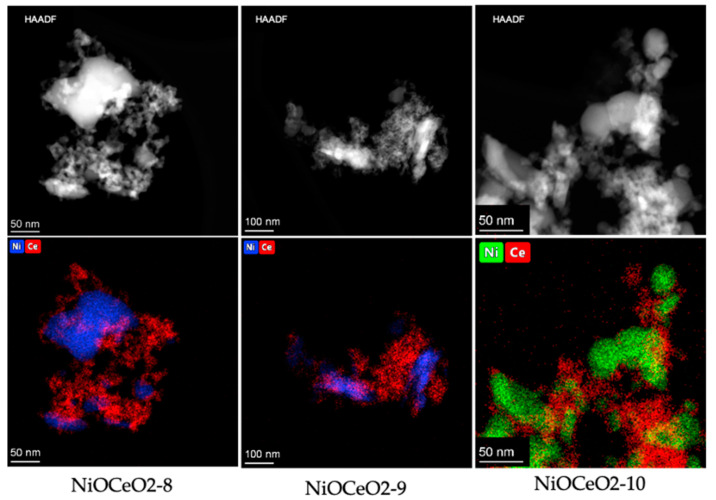
HAADF images and EDS mappings corresponding to the reduced NiOCeO_2_-8 (**left**), NiOCeO_2_-9 (**center**) and NiOCeO_2_-10 (**right**) catalysts.

Complementing the chemisorption results with others obtained using more direct techniques, such as electron microscopy, provides a more realistic description of the materials studied, in terms of the arrangement of the CeO_2_ and NiO (Ni) phases. The results obtained in the electron microscopy characterization of the samples are displayed in Figure 3, Figure 4 and Figure 5.

Figure 3 shows a representative TEM image of the as-prepared NiO-CeO_2_ mixed oxides, in which NiO was found as plate-like particles and CeO_2_ appeared to form small crystals with a size of about 5 nm. The arrangement of the prepared systems would thus differ from the classical ones characterized by the existence of NiO particles dispersed on the surface of the oxide used as a support (CeO_2_). The reflections observed in the DDPs (shown as insets) were indexed on the basis of cubic (NiO) and fluorite (CeO_2_) structures, respectively, in close agreement with the results obtained by XRD.

After reduction treatment at 650 °C in H_2_(5%)/Ar, Ni appears as crystallites with different sizes, in some cases reaching values of around 40–50 nm, as shown in Figure 4, for samples NiOCeO_2_-8 and NiOCeO_2_-10. In order to better identify the different phases and spatial distribution of Ni and Ce, HAADF-STEM images and composition STEM-EDS mappings have been recorded (Figure 5).

Since the intensity of the HAADF-STEM images is proportional to the square of the atomic number of the element, the brightest areas in these images should correspond to the presence of Ce (Z = 58), and those of less intensity should correspond with the presence of Ni (Z = 28). However, because this intensity also depends on the thickness of the sample, the difference in contrast is not always indicative of the element’s distribution. Therefore, the corresponding STEM-EDS maps for the areas observed in the HAADF-STEM images have also been recorded. The combination of both techniques reveals the existence of Ni aggregates of different sizes and morphologies surrounded by CeO_2_. The images confirm the presence of Ni particles with a high heterogeneity in size, although the limited number of images recorded did not permit reliable size distributions to be obtained. It should be pointed out that, according to the results derived from H_2_-chemisorption studies, these distributions would be shifted towards larger particle size values in the case of sample NiOCeO_2_-10.

### 3.2. Catalytic Activity of NiOCeO_2_-pH in CO Methanation

**Figure 6:**
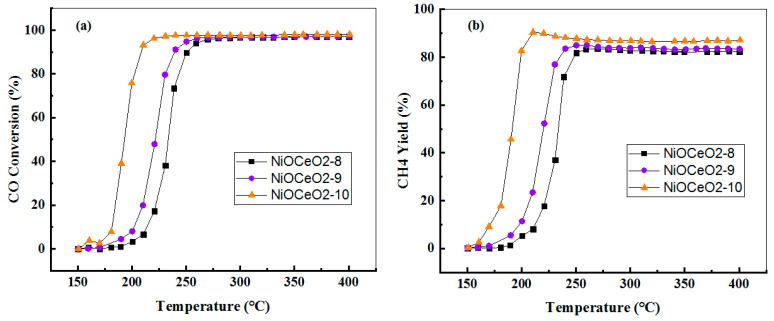
Catalytic performance of NiOCeO_2_ in CO methanation as a function of temperature: (**a**) CO conversion and (**b**) CH_4_ yield.

The NiOCeO_2_ catalysts were tested in the catalytic CO methanation reaction. The CO conversion and CH_4_ yield curves are displayed in Figure 6. As can be deduced from this figure, there is a remarkable difference in the evolution of the curves, thus evidencing the effect of the synthesis pH on the catalytic performance of these materials. Both CO conversion and CH_4_ yield improve significantly as the pH of the synthesis increases from 8 to 9, and even more markedly from 9 to 10. This effect can be clearly evidenced from the values of temperature required to achieve a 50% CO conversion, which are: 232, 225 and 184 °C for samples NiOCeO_2_-8, NiOCeO_2_-9 and NiOCeO_2_-10, respectively. The CO conversion obtained with the NiOCeO_2_-10 catalyst at 235 °C was almost complete, while for the NiOCeO_2_-8 and NiOCeO_2_-9 catalysts it was necessary to increase the temperature up to 280 °C to achieve maximum conversion values. Similar differences were found in terms of CH_4_ yield, always indicating the superior response of the NiOCeO_2_-10 catalyst. For a better comparison of the capacity of the three catalysts to activate the reaction, and also with the aim of correcting for the effect of Ni loading (slightly higher in the case of the sample prepared at pH = 10 according to ICP results), the CO conversion rate and TOF values at low temperature (220 °C) have been calculated (Table 3).

The results shown in Table 3 indicate that when the activity is expressed in terms of Ni mass (or Ni atoms) the differences between the catalysts are even more pronounced. An increase in conversion rates by a factor of ~3 is observed between NiOCeO_2_-8 and NiOCeO_2_-9 and by a factor of 5 between NiOCeO_2_-8 and NiOCeO_2_-10. Differences in terms of TOF have also been estimated, showing the same trend.

The best performance is obtained with the sample exhibiting the lowest Ni dispersion. This finding agrees with those reported by other authors and points to the already reported structure-sensitive character of CO methanation. For example, Takenaka et al. reported that Ni metal particles with relatively large diameters (about 20−100 nm) were more active in the CO methanation reaction [8]. Aksoylu et al. also investigated structure/activity relationships in coprecipitated nickel-alumina catalysts using CO_2_ adsorption and methanation. They found that the higher catalytic activity was obtained at high Ni loadings due to the formation of large Ni particles [29]. Gao et al. compared the performance of Ni/Al_2_O_3_ and Ni/BHA (BHA: barium hexaaluminate) catalysts, with the same loading, for the production of SNG via CO methanation reaction. The presence of larger Ni particles (20–40 nm) is proposed as one of the explanations for the superior performance of the Ni/BHA catalyst [30].

Beyond metallic dispersion, the textural properties of catalysts (mainly total specific surface area and porosity) have been shown to play an important role in catalysis. In this sense, we must recall that one of the effects of pH on the properties of the oxides was the increase of the specific surface area as the pH of the synthesis increased. In addition, we should also mention the differences observed in the pore-size distribution curves. The NiOCeO_2_-10 catalyst showed a more homogeneous porosity, characterised by a narrow peak below 40 nm, with a rather small contribution of larger diameter pores. On the other hand, samples prepared at pH 8 and 9 showed a more heterogeneous porosity, with a main contribution around 30–50 nm and a higher relevance of larger pores (>60 nm). Therefore, the effect of these textural properties on the superior performance of NiOCeO_2_-10 cannot be ruled out, in close agreement with the results reported by Le et al. [5]. These authors evaluated the catalytic performance for CO and CO_2_ methanation over Ni/CeO_2_ catalysts with different surface areas and concluded that the catalytic activities increased with increasing CeO_2_ surface area.

**Figure 7:**
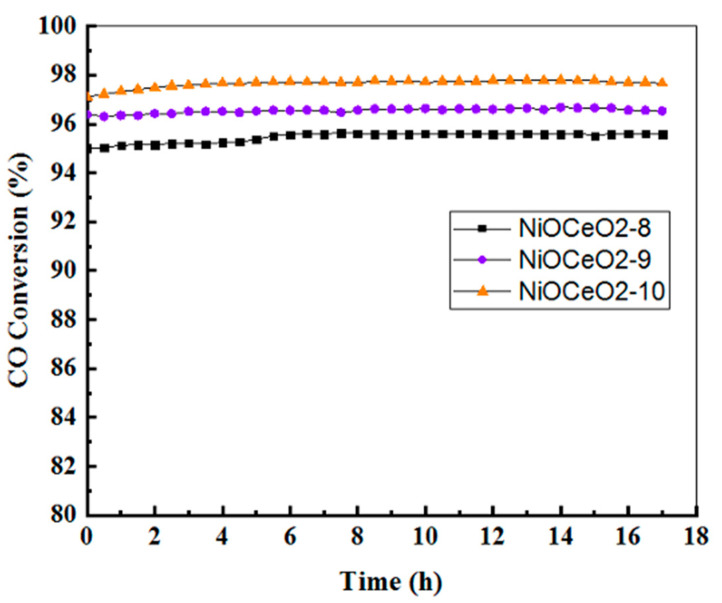
Stability tests for CO methanation at 250 °C.

Catalytic stability was evaluated at a fixed temperature (T = 250 °C) during 17 h of time on stream. This temperature was selected because it was the minimum temperature at which the respective highest conversions were simultaneously achieved for the three samples. Figure 7 shows CO conversion curves as a function of time. As can be seen, excellent stability was obtained with the NiOCeO_2_ catalysts, regardless of the synthesis conditions.

## 4. Conclusions

A series of NiOCeO_2_-pH (pH = 8, 9 and 10) mixed oxides was successfully prepared by a variation of the hydrotalcite method. The catalytic activity of these catalysts for CO methanation was investigated. NiOCeO_2_-pH mixed-oxide catalysts exhibited different physicochemical properties, resulting in catalysts with high activity at low temperature and outstanding stability at high temperature, in CO methanation. It was found that the catalytic activity increased when increasing the pH at which the samples were prepared, with the NiOCeO_2_-10 catalyst being the most active. The influence of this synthetic parameter on textural properties (higher pH results in higher surface area, narrower porosity distribution and larger Ni particle size) seems to be the reason for such behavior.

The results reported and the conclusions derived from them illustrate the correlation between the three basic axes in materials science: (i) preparation methods (and influence of synthetic variables), (ii) physicochemical properties of the materials obtained and (iii) performance. This correlation is of primary importance at the academic level, and also constitutes the basis for the design of new advanced and efficient materials for industrial applications.

## Figures and Tables

**Table 1:** Structural and textural properties (XDR data, Ni content (ICP), BET surface area, pore volume, pore diameter) of NiOCeO_2_ catalysts after calcination at 600 °C.

Samples	Crystalline Phases	Average Crystallite Size (nm)	Lattice Parameter (Å)	Ni (wt%)	S_BET_ (m^2^/g)	V_P_ (cm^3^/g)	D_P_ (nm)
CeO_2_-8	CeO_2_-fluorite	12.6	5.410	--	--	--	--
NiOCeO_2_-8	NiO-cubic CeO_2_-fluorite	18.7 5.2	4.170 5.397	28.0	73	0.47	25.6
NiOCeO_2_-9	NiO-cubic CeO_2_-fluorite	14,3 4,2	4.173 5.399	28.0	80	0.60	30.3
NiOCeO_2_-10	NiO-cubic CeO_2_-fluorite	13.0 4.8	4.169 5.405	32.2	91	0.44	19.6

**Table 2:** H_2_-Chemisorption analysis of reduced NiOCeO_2_ catalysts.

Sample	Ni Dispersion (%)	Ni Particle Size (nm)	Ni Surface Area (m^2^/g)
NiOCeO_2_-8	8.1	12.5	15.1
NiOCeO_2_-9	7.7	13.2	14.3
NiOCeO_2_-10	2.4	41.0	4.6

**Table 3:** Catalytic results at 220 °C.

Catalysts	CO Conversion (%)	Conversion Rate (mmolCO·g_Ni_^−^^1^·h)	TOF (s^−1^) × 10^3^
NiOCeO_2_-8	16.7	2.9	5.9
NiOCeO_2_-9	48.3	8.4	17.9
NiOCeO_2_-10	96.7	14.8	101.3
